# *BMC Medical Informatics and Decision Making* reviewer acknowledgement 2015

**DOI:** 10.1186/s12911-016-0261-z

**Published:** 2016-02-22

**Authors:** Giulia Mangiameli

**Affiliations:** BioMed Central, Floor 6, 236 Gray’s Inn Road, London, WC1X 8HB UK

## Abstract

The editors of *BMC Medical Informatics and Decision Making* would like to thank all our reviewers who have contributed to the journal in Volume 15 (2015)

**Jos Aarts**

Netherlands

**Dionisio Acosta**

UK

**Leila Ahmadian**

Iran

**Larry Allen**

USA

**Hussam Alshraideh**

Jordan

**Milena Anatchkova**

USA

**Jessica Ancker**

USA

**Shilo Anders**

USA

**Giuseppe Andreoni**

Italy

**Steven Babin**

USA

**Mirza Baig**

New Zealand

**Charles Baillie**

USA

**Gianluca Baio**

UK

**Erin Bantum**

USA

**Giulia Barbati**

Italy

**Laura Barnes**

USA

**Melissa Baysari**

Australia

**Jeff Belkora**

USA

**Brian Bell**

UK

**Barbara Bennett**

Australia

**Corinna Bergelt**

Germany

**Marnie Bertolet**

USA

**Richard Birtwhistle**

Canada

**Seiji Bito**

Japan

**Jennifer Boger**

Canada

**Carissa Bonner**

Australia

**Timothy Bonnici**

UK

**Damian Borbolla**

USA

**Simone Borsci**

UK

**Daniel Bossen**

Netherlands

**Jacques Bouaud**

USA

**Rick Bradshaw**

USA

**Olivier Johan Tavai Briët**

Switzerland

**Benjamin Brown**

UK

**Angela Buchholz**

Germany

**Christopher Buckingham**

UK

**Teresa Burgess**

Australia

**Harry Burke**

USA

**Erik Cambria**

Singapore

**Manuel Campos**

Spain

**Dongsheng Cao**

China

**Fran Carroll**

UK

**German Castellanos-Dominguez**

Colombia

**Shaneez Chatharoo**

UK

**Niels Chavannes**

Netherlands

**You Chen**

USA

**Rie Chiba**

Japan

**Deena Chisolm**

USA

**Alan Chiu**

USA

**Songphan Choemprayong**

Thailand

**Chun-An Chou**

USA

**Yiing-Jenq Chou**

Taiwan

**Jean-Paul Chretien**

USA

**Katerina Christopoulos**

USA

**Theopisti Chrysanthaki**

UK

**Michele Ciulla**

Italy

**Robyn Clay-Williams**

Australia

**Sean Coady**

USA

**Trevor Cohen**

USA

**Tiago Colicchio**

USA

**Tracy Comans**

Australia

**Pascal Coorevits**

Belgium

**Erdal Cosgun**

Turkey

**Catherine Craven**

USA

**Caroline Crawford**

USA

**Kathrin Cresswell**

UK

**Kris Cuppens**

Belgium

**Wenrui Dai**

China

**Antonio D'Ambrosio**

Italy

**Gianfranco Damiani**

Italy

**Abhijit Dasgupta**

USA

**Martin Dawes**

Canada

**Theodore Day**

USA

**Derek De Beurs**

Netherlands

**Jeroen De Bruin**

Austria

**Sarah Deeny**

UK

**Guilherme Del Fiol**

USA

**Spiros Denaxas**

UK

**Don Detmer**

USA

**Haryana Dhillon**

Australia

**Birthe Dinesen**

Denmark

**Brian Dixon**

USA

**Cristian Dogaru**

UK

**David Dorr**

USA

**Adam Dunn**

Australia

**Claudio Eccher**

Italy

**Mikel Egana Aranguren**

Canada

**Birgit Eiermann**

Sweden

**Sylvia Elkhuizen**

Netherlands

**Mortada El-Shabrawi**

Egypt

**Nadiye Erdil**

USA

**Miguel Angel Fernandez Granero**

Spain

**Jesualdo Tomas Fernandez-Breis**

Spain

**Carlos Fernandez-Llatas**

Spain

**Rama Flarsheim**

Canada

**Jose F Florez-Arango**

Colombia

**Randi Foraker**

USA

**Daniel Fort**

USA

**Allison Foster**

USA

**Paolo Fraccaro**

UK

**Anneke Francke**

Netherlands

**Glaura Franco**

Brazil

**Emma Frew**

UK

**Rocco Friebel**

UK

**Kevin Fuij**

USA

**Marie-Pierre Gagnon**

Canada

**Xiaohong Gao**

UK

**Ana García-Bernabeu**

Spain

**Maria Garcia-Gil**

Spain

**Jennifer Garvin**

USA

**Susanne Georgsson Öhman**

Sweden

**Daniele Giansanti**

Italy

**Michael Gionfriddo**

USA

**James Giordano**

USA

**William Goggins**

Hong Kong

**Virgilio Gomez Rubio**

Spain

**William Goossen**

Netherlands

**Kathleen Gray**

Australia

**Zachary Grinspan**

USA

**Stephanie Haas**

USA

**John Halamka**

USA

**Ronald Halbert**

USA

**Andrew Hall**

UK

**David Hanauer**

USA

**Ron Leonardus Hubertus Handels**

Netherlands

**Frank Harrell**

USA

**Peter Haug**

USA

**Lotte Haverman**

Netherlands

**Sarah Hawley**

USA

**Harald Heinzl**

Austria

**Michelle Hendriks**

Netherlands

**Vitaly Herasevich**

USA

**Antonio Hernández Niñirola**

Spain

**Patrick Hester**

USA

**Harry Hochheiser**

USA

**Jason Hockenberry**

USA

**Jacob Hofdijk**

Netherlands

**Bree Holtz**

USA

**Lars Hölzel**

Germany

**Peter Hoonakker**

USA

**Susan Hrisos**

UK

**William Hsu**

USA

**Bernice Huang**

USA

**Ursula Hübner**

Germany

**J. Marjan Hummel**

Netherlands

**Raul Igual**

Spain

**Piper Jackson**

Canada

**Guoqian Jiang**

USA

**Constance Johnson**

USA

**Rachel Johnson**

UK

**Jaranit Kaewkungwal**

Thailand

**Charles Kahn**

USA

**Minna Kaila**

Finland

**Chandrakar Kamath**

India

**Hong Kang**

USA

**Garehatty Kanthraj**

India

**David Kaufman**

USA

**Karen Kelly-Blake**

USA

**Ahsan Khandoker**

United Arab Emirates

**Mehdi Khashei**

Iran

**Patrick Kierkegaard**

UK

**Ho Kim South**

Korea

**Jeffrey Klann**

USA

**Ela Klecun**

UK

**Peter Knapp**

UK

**Peter Kokol**

Slovenia

**Bevan Koopman**

Australia

**Georgy Kopanitsa**

Russian Federation

**Ioannis Korkontzelos**

UK

**Grigorios Kotronoulas**

UK

**Markus Kreuzthaler**

Austria

**Levente Kriston**

Germany

**Anand Kulanthaivel**

USA

**Craig Kuziemsky**

Canada

**Hallvard Laerum**

Norway

**Uttama Lahiri**

India

**Pauline Siew Mei Lai**

Malaysia

**Douglas Lake**

USA

**Deirdre Lane**

UK

**Annie Lau**

Australia

**Yvon Lebranchu**

France

**Tian-Fu Lee**

Taiwan

**Young Ji Lee**

USA

**France Légaré**

Canada

**Pascale Lehoux**

Canada

**Lasse Lensu**

Finland

**Hugo Leroux**

Australia

**M. Leu**

USA

**Jörg D. Leuppi**

Switzerland

**Haomin Li**

China

**Linda Li**

Canada

**Wenmin Li**

China

**Yong Li**

China

**Zhongjie Li**

China

**Valentina Lichtner**

UK

**Sarah Lillie**

USA

**Ellen Lipstein**

USA

**Nan Liu**

Singapore

**Zengjian Liu**

China

**Miguel López-Coronado**

Spain

**Karen Lorimer**

UK

**Jake Luo**

USA

**Yashar Maali**

Australia

**Giovanni Maccioni**

Italy

**Lynette Mackenzie**

Australia

**Nicola Mackintosh**

UK

**Vijay Mago**

Canada

**Jose Alberto Maldonado**

Spain

**Ben Marafino**

USA

**Mar Marcos**

Spain

**Chris Martin**

UK

**Catalina Martínez**

Costa Austria

**Barbara Massoudi**

USA

**J. Mac Mccullough**

USA

**Michelle Mcdowell**

Germany

**Brian Mckinstry**

UK

**Amaia Mendez Zorrilla**

Spain

**Jane Messina**

USA

**Margreet Michel-Verkerke**

Netherlands

**Armin R. Mikler**

USA

**Laura Militello**

USA

**Dragana Miljkovic**

Slovenia

**Anne Miller**

Australia

**Victor Minichiello**

Australia

**Riccardo Miotto**

USA

**Jose Joaquin Mira**

Spain

**Alin Moldoveanu**

Romania

**Linda Moniz**

USA

**Roger Morbey**

UK

**Sandra Morelli**

Italy

**Hamid Mukhtar**

Saudi Arabia

**Adolfo Muñoz Carrero**

Spain

**Radhakrishnan Nagarajan**

USA

**Kim Nazi**

USA

**Aurelie Neveol**

France

**Axel Newe**

Germany

**Huong Nguyen**

USA

**Stuart Nicholls**

Canada

**Edward Nicol**

South Africa

**Mehrbakhsh Nilashi**

Malaysia

**Angelo Nuzzo**

Austria

**Mary Ann O'Brien**

Canada

**William Ogallo**

Kenya

**Hans Ossebaard**

Netherlands

**Casey Overby**

USA

**Zenggang Pan**

USA

**Chris Paton**

UK

**Marco Paulli**

ITALY

**Philip Payne**

USA

**Leandro Pecchia**

UK

**Linda Peelen**

Netherlands

**Robert Penfold**

USA

**David Perez-Rey**

Spain

**Lionel Perrier**

France

**Rimma Pivovarov**

USA

**Petra Povalej Brzan**

Slovenia

**John Powell**

UK

**Mattia Prosperi**

USA

**Siyu Qian**

Australia

**Preethi Raghavan**

USA

**Rebecca Randell**

UK

**Luke Rasmussen**

USA

**Raj Ratwani**

USA

**Pradeep Ray**

Australia

**Rachel Richesson**

USA

**Britta Ricker**

USA

**Greg Roberts**

Australia

**Kirk Roberts**

USA

**Álvaro Rocha**

Portugal

**Pedro Pereira Rodrigues**

Portugal

**Lionel Rostaing**

France

**Robert Rudin**

USA

**Pekka Ruotsalainen**

Finland

**Mandy Ryan**

UK

**Lucia Sacchi**

Italy

**Satya Sahoo**

USA

**Farrant Sakaguchi**

USA

**Frank Sandmann**

UK

**Reidun Sandvik**

Norway

**Silvina Santana**

Portugal

**Nicola Saplding**

UK

**Philip Scott**

UK

**Karen Sepucha**

USA

**Walter Sermeus**

Belgium

**Berkan Sesen**

UK

**Aviv Shachak**

Canada

**Nigam Shah**

USA

**Megan Shen**

USA

**Soo-Yong Shin**

South Korea

**Mauro Silvestrini**

Italy

**Sonal Singh**

USA

**Peter Singleton**

UK

**Sam Smith**

UK

**Parikshit Sondhi**

USA

**Joao Sousa**

Portugal

**Ross Sparks**

Australia

**Matthew Sperrin**

UK

**Ton Spil**

Netherlands

**Stephen Andrew Spooner**

USA

**Cord Spreckelsen**

Germany

**Douglas Steinke**

UK

**Gregor Stiglic**

Slovenia

**Ellen Stuart**

Ireland

**Abdülhamit Subaşı**

Bosnia and Herzegovina

**Xing-Guo Sun**

China

**Michael Swash**

UK

**Stephen Swift**

UK

**Ricky Taira**

USA

**Anhui Tan**

China

**Derek Tang**

USA

**Huibert Tange**

Netherlands

**Cui Tao**

USA

**Alan Taylor**

Australia

**Darlene Taylor**

Canada

**Johanna Taylor**

UK

**Richard Thomson**

UK

**Thankam Thyvalikakath**

USA

**Binyam Tilahun**

Ethiopia

**Salvador Tortajada**

Spain

**Monica Tremblay**

USA

**Athanasios Tsanas**

UK

**Samson Tu**

USA

**Francesco Turturro**

USA

**Alexandros Tzallas**

Greece

**Rupa Valdez**

USA

**Vanya Van Belle**

Belgium

**Peter Van Der Putten**

Netherlands

**Heleen Van Der Sijs**

Netherlands

**Mariette Van Engen-Verheul**

Netherlands

**Tjeerd Van Staa**

UK

**Gonzalo Vazquez-Prokopec**

USA

**Aman Verma**

Canada

**Karin Verspoor**

Australia

**Susanne Vijverberg**

Netherlands

**David Vivas-Consuelo**

Spain

**Silke Von Esenwein**

USA

**Frans Voorbraak**

Netherlands

**Victoria Anne Wade**

Australia

**Fei Wang**

USA

**Jing Wang**

USA

**Jeremy Warner**

USA

**Marieke Weernink**

Netherlands

**Charlene Weir**

USA

**James Weisfeld-Adams**

USA

**Jobke Wentzel**

Netherlands

**Perla Werner**

Israel

**Susan White**

USA

**James Whyte**

USA

**Michael Wiegand**

Germany

**Jane Wilcox**

UK

**Chris Williams**

UK

**Qi Xie**

China

**Lei Yang**

China

**Po-Yin Yen**

USA

**Meliha Yetişgen**

USA

**Tracey Young**

UK

**Ping Yu**

Australia

**Konstantinos Zacharias**

UK

**Fengqing Zhang**

USA

**Kui Zhang**

USA

**Pei Zhang**

USA

**Ping Zhang**

USA

**Rui Zhang**

USA

**Wei Zhang**

USA

**Zhen Zhang**

USA

**Guoru Zhao**

China

**Wojciech Ziarko**

Canada

**Jördis Maria Zill**

Germany

**Kate Zinszer**

Canada

